# Liver Transplant in Patients with Hepatocarcinoma: Imaging Guidelines and Future Perspectives Using Artificial Intelligence

**DOI:** 10.3390/diagnostics13091663

**Published:** 2023-05-08

**Authors:** Mihai Dan Pomohaci, Mugur Cristian Grasu, Radu Lucian Dumitru, Mihai Toma, Ioana Gabriela Lupescu

**Affiliations:** 1 Department of Radiology and Medical Imaging, Fundeni Clinical Institute, 022328 Bucharest, Romania; pomohaci0mihai@gmail.com (M.D.P.); radu.dumitru@gmail.com (R.L.D.); tomamihai@gmail.com (M.T.); ilupescu@gmail.com (I.G.L.); 2 Department of Radiology, The University of Medicine and Pharmacy “Carol Davila”, 050474 Bucharest, Romania

**Keywords:** hepatocarcinoma, cirrhosis, liver transplantation, liver transplant, artificial intelligence, machine learning, radiomics, deep learning, neural networks

## Abstract

Hepatocellular carcinoma is the most common primary malignant hepatic tumor and occurs most often in the setting of chronic liver disease. Liver transplantation is a curative treatment option and is an ideal solution because it solves the chronic underlying liver disorder while removing the malignant lesion. However, due to organ shortages, this treatment can only be applied to carefully selected patients according to clinical guidelines. Artificial intelligence is an emerging technology with multiple applications in medicine with a predilection for domains that work with medical imaging, like radiology. With the help of these technologies, laborious tasks can be automated, and new lesion imaging criteria can be developed based on pixel-level analysis. Our objectives are to review the developing AI applications that could be implemented to better stratify liver transplant candidates. The papers analysed applied AI for liver segmentation, evaluation of steatosis, sarcopenia assessment, lesion detection, segmentation, and characterization. A liver transplant is an optimal treatment for patients with hepatocellular carcinoma in the setting of chronic liver disease. Furthermore, AI could provide solutions for improving the management of liver transplant candidates to improve survival.

## 1. Introduction

According to the Global Cancer Observatory, primary liver cancer is ranked the third most frequent cause of death and the sixth most commonly diagnosed cancer in 2020 [1]. Hepatocellular carcinoma (HCC) is the most common type of primary liver cancer, accounting for approximately 75–85% of cases [2], representing a significant public health burden worldwide. The incidence of HCC is most often linked with chronic liver disease, and cirrhosis is the primary risk factor, with one-third of cirrhotic patients reported to develop liver cancer during their lifetime [2]. The most common cause of chronic liver disease in Europe is the hepatitis C virus, followed by excessive alcohol intake [3]. In addition, there is a male predominance compared to women of 2:1 [2].

While reducing the incidence of chronic viral hepatitis remains an important goal to prevent the development of chronic liver disease and HCC, other nonviral risk factors besides alcohol consumption are emerging as public health issues in developed countries. Non-alcoholic fatty liver disease (NAFLD) is reported as the most common cause of hepatic dysfunction worldwide [4], with a prevalence of 25–25% in the general population [5], and it is projected to reach around 33.5% in 2030 [6]. Together with non-alcoholic steatohepatitis (NASH), they further influence the development of HCC [2] as these two entities have a similar potential to progress to advanced liver fibrosis [4].

HCC has been the main indication for transplantation in patients with oncologic disease. Together with NASH and NAFLD, it is described as the fastest-rising indication for hepatic transplant [7]. In theory, it is the optimal treatment option because it has a double role of eliminating the underlying liver disease while removing the lesion [8]. However, the selection of transplant candidates that have developed HCC needs to be rigorous as there is a general organ shortage. The United Network for Organ Sharing (UNOS) has described a drop-out of 20% in patients awaiting transplantation [9]. Therefore, extended donor criteria have been adopted to reduce these figures, like older donors, fatty liver, and cardiac arrest donors with inevitable inferior post-transplant outcomes [9]. These factors further stress the importance of patient selection and organ allocation to reduce mortality and improve post-transplantation survival.

**Figure 1:**
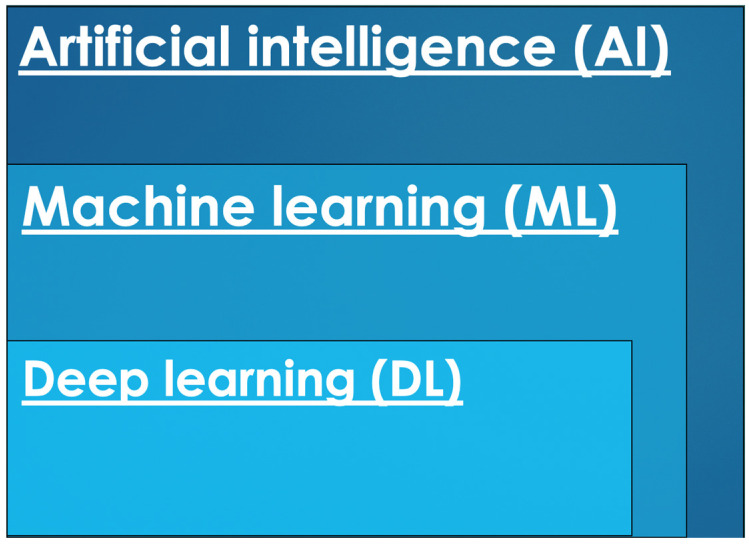
Simplified graphical representation of artificial intelligence and its subsets.

The demand for precision medicine and personalized treatments, together with technological advances, has led to an increasing amount of research regarding the application of artificial intelligence (AI) to medical images. The term and technology are not new, as the first artificial neuron was described in 1943 [10]. Today, AI is a large field of study incorporating algorithms capable of solving tasks that normally require human intelligence. Machine learning (ML) is a subset of AI that involves extracting patterns from data without explicit programming [11]. As the algorithm has a more complex structure with multiple components, or “layers”, the term deep learning (DL) is used as a subset of ML [11]. A simplified version of the relationship between these AI divisions is presented in Figure 1, with DL being included in the ML category and both types of AI technologies.

**Figure 2:**
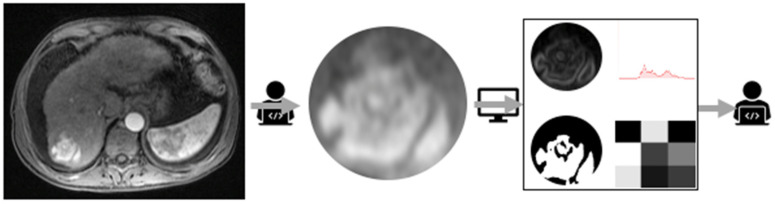
Simplified radiomics model workflow.

Radiomics is a type of ML that has gained attention because it can extract complex imaging data that could reflect the underlying biological properties of tissues [12]. The algorithm can obtain quantitative features like histogram, shape, texture, radial and transform-based characteristics which are too detailed for normal human vision to analyse [13]. These extracted features are analysed by researchers using other AI techniques, and the most relevant ones are chosen for implementation [13]. A simplified radiomics model workflow is portrayed in Figure 2. The user first processes the data, the features are extracted by the ML model, and then the features are selected by the user using a variety of techniques.

**Figure 3:**
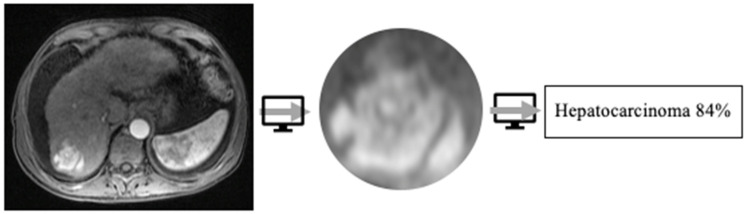
Simplified DL model workflow.

Convolutional neural networks (CNN) represent one of the most successful types of DL algorithms that work explicitly with images and have great potential in radiology [14]. Compared to radiomics, which needed a “human-in-the-loop” approach to analysing the features, CNNs provide a more “end-to-end” approach as they can segment, analyse, and provide output without human intervention [15]. A simplified DL model workflow is portrayed in Figure 3. Compared to the previous radiomics model, the DL algorithm can process the data and automatically choose the most relevant features.

The advent of Electronic Health Records (EHR) and digital imaging has led to an increase in medical data with an estimated annual growth of 48% from 2013 to 2020 [16]. The amount of annually increasing data in radiology makes it a main field of application for these algorithms, which promise to alleviate the imaging burden and help provide better patient care. Also, because medical images contain a lot of embedded information, there is hope that more quantitative data can be extracted at a voxel-wise level for diagnostic, staging and prediction purposes. 

Several AI models have been tested on clinical and laboratory data to better stratify organ allocation strategies and graft matching [17,18,19]. In addition, it has been stated that its dynamic properties regarding testing and validation allow it to better adapt to different populations [20]. 

Generally, four primary tasks can be pursued in medical image analysis and interpretation: classification, localization, detection, and segmentation [21]. Classification involves assigning a label to the image (e.g., hepatocellular carcinoma, cholangiocarcinoma, haemangioma, etc.). Localization and detection involve the application of bounding boxes to the structures or lesions of interest and are often a preliminary step to other functions. Finally, segmentation is a complex task as it assigns pixels to a class (e.g., lesion) in a given image to create precise boundaries from surrounding tissues (e.g., liver).

The abdominal region was ranked third in applying DL to radiology in an 8-year timespan between 2012 and 2020 [22]. A more focused review of AI hepatic imaging applications ranked diagnosis as the most researched function, followed by prognosis and segmentation. Also, HCC was the most common research interest [23]. However, the number of clinically approved AI applications is limited, caused by the lack of external and prospective validation and limited well-curated and available datasets. Furthermore, the number of clinically approved hepatic algorithms is inferior to other organs and structures, with only two applications described in an analysis of 100 commercially available radiology products [24]. 

Our objectives are to review the current imaging protocols and guidelines for liver transplantation in the setting of HCC and to do an overview of emerging AI applications that can be applied for better patient management.

## 2. Liver Transplant in HCC

In the following section, we provide a brief review of imaging protocols and guidelines for liver transplantation in the setting of HCC. 

A liver transplant is an optimal treatment for patients with HCC and cirrhosis because it targets the underlying disease and the tumour [8]. However, the patients eligible for this treatment must be carefully selected because there is a general organ shortage, and these patients will go through lifelong immunosuppression. 

The most widely used criteria for orthotopic liver transplant (OLT) selection in patients with HCC are the Milan criteria [25], developed in 1996. They are recommended by the European Association for the Study of the Liver (EASL) [26], the European Society of Medical Oncology (ESMO) [27], the National Comprehensive Cancer Network (NCCN) [28] and the American Association for the Study of Liver Diseases (AASLD) [29]. According to Milan, LT is recommended in patients with one lesion less than or equal to 5 cm or up to 3 lesions, each less than or equal to 3 cm. Using these criteria, the five-year survival rates are 65–80% [26]. Because they delineate a group of patients with cirrhosis and HCC that have transplant results similar to those only with cirrhosis, they have been included since 2002 in UNOS. This organisation handles organ transplants in the USA. These criteria apply to patients unsuitable for resection, often because of advanced underlying hepatic disease. Extrahepatic disease or vascular tumour invasion are absolute contraindications to LT [26,27,28]. 

Living donor liver transplant is not very popular in Europe, as it represents around 6–7% of all LT performed yearly, according to data from Euro-transplant statistics in 2020–2021 [30]. However, marginal grafts remain an option that can be applied in selected patients and centres with experience [26,27]. 

## 3. Extending Milan

With LT being the therapy with the highest probability of curing HCC [31], a lot of research has been done to find the best solution to extend the Milan criteria and to find new markers that better stratify patients to improve the selection of candidates for this treatment option [32,33,34,35,36,37,38,39,40]. All these criteria describe an extrahepatic disease or macrovascular tumour invasion as absolute contraindications to LT. A summary of transplant criteria is provided in Table 1. 

Several studies have investigated the relevance of alpha-fetoprotein (AFP) tumour markers in managing these patients [41,42] with a higher risk of recurrence in patients with high AFP levels. Thus, it has been included in the Metro ticket V2.0, AFP model, and Hangzhou criteria, and a threshold of 1000 ng/dL is currently applied in the UNOS criteria.

Other criteria emerge that try to include more robust data like histopathological information, including tumour differentiation in the Extended Toronto criteria and the evaluation of tumour grading in the Hangzhou criteria. Volumetric information can also be used since lesions can sometimes have a variable shape. Thus, a threshold criterion has been developed in the TTV criteria. 

While the Milan criteria remain the most widely recommended in the international guidelines, national policies have also allowed the adoption of other models [43]. For example, the AFP model has been used in France since 2012. In addition, the Milan, UCSF, TTV, Up-to-7 criteria, and the AFP model are all accepted in Italy. In Spain, both Milan and Up-to-7 criteria are used.

To increase the chance for transplant in patients with HCC, the use of loco-regional treatments is supported either to reduce the risk of drop-out in patients within Milan criteria (“Bridging”) or to downstage patients beyond Milan criteria [26,27,28]. The response to loco-regional treatments can be used as a marker for transplant outcome prediction [44], and it has been included in the TRAIN criteria [40] because a good response is associated with less probability of microvascular invasion or low tumour grading [44]. The TRAIN score also proved to be the best predictor for microvascular invasion.

Although a consensus on the best option for expanding the LT criteria has not been met, biological and dynamical markers will likely replace morphological data [45]. In this context, AI is a potential aid to process complex information, better stratify these patients and provide AI markers that reflect tumour biology and aggressiveness. 

**Figure 4:**
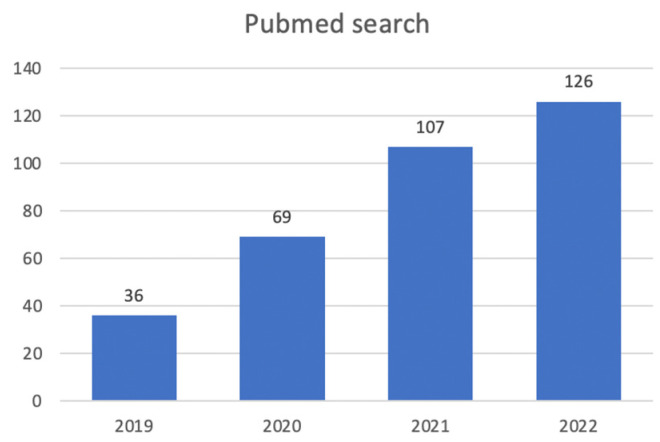
A PubMed search for liver transplant and AI.

The graph below shows an apparent increase in the number of articles regarding AI solutions in hepatic transplants (Figure 4). The figure plots data from 2019 to 2022 obtained from PubMed using the ‘Advanced search’ and applying the keywords “Liver transplant”, “Artificial intelligence”, “Machine learning”, “Neural networks”, and Boolean operators AND, OR. The results represent the number of papers published per year with the aforementioned filters.

## 4. AI-Aided Evaluations in Candidates for LT with HCC

The following will review the most critical AI applications that might impact transplant imaging.

### 4.1. Detection

Detection involves applying a bounding box to the region of interest in the processed images (e.g., lesions, organs, etc.). It is often a preliminary step of more complex algorithms that use a combination of detection-segmentation classification.

Major international guidelines recommend ultrasound (US) as the main imaging surveillance tool for patients with cirrhosis [26,29]. US has a detection sensitivity for HCC of around 84%, but with a substantial drop for early-stage lesions, to almost 47% [46]. This is essential as these lesions have the highest likelihood of long-term cures using radical treatments [8]. However, contrast-enhanced (CE) CT and MRI are not cost-effective for the general surveillance of HCC, except for patients awaiting transplants, according to the EASL guidelines [26]. Therefore, CT is more widely used as it has lower costs, faster acquisition times and less susceptibility to motion artifacts. However, it uses ionizing radiation with lower soft tissue contrast [47]. Although MRI has increased costs and acquisition times, it offers superior tissue contrast and can also use hepatospecific contrast agents, increasing sensitivity [47].

An AI-detection tool would be essential in monitoring patients on the transplant list since the presence of HCC implies the accordance with MELD (Model for End-Stage Liver Disease) exception points [48], which changes the prioritisation on the transplant list and can permit an earlier treatment. Furthermore, the importance of early detection cannot be overstated, as it can impact survival. This is relevant as more patients will be identified with smaller lesions within Milan criteria, with favourable 5-year survival rates of around 65–80% [26]. Thus, an integrated AI tool for lesion detection could improve diagnostic accuracy and improve transplant patient stratification. The summary of the AI-detection models presented below is shown in Table 2.

**Figure 5:**
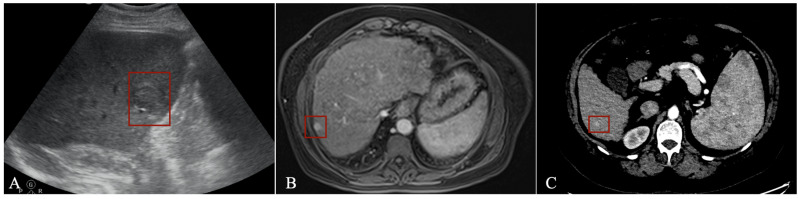
Detection algorithm using ultrasound (**A**), MRI (**B**) and CT (**C**) imaging in the arterial phase.

In US imaging, the number of applications dedicated to focal liver lesions is reduced mainly due to limited datasets available and because liver lesion characteristics often overlap [49]. However, such a tool can aid those performing US examinations, especially in centres with limited experience. Tiyarattanachai et al. [50] prospectively evaluated US images from 334 patients using a RetinaNet DL model, obtaining detection rates for focal liver lesions as high as 89.8%, surpassing that of clinicians. For HCC, the detection rate was 100%, but only 23 cases were included in the study. Lee et al. [51] used a CNN to detect HCC in multiphase CECT imaging from 302 CT studies using all three phases (arterial, venous, and delayed) with a sensitivity of 93.88%. Using multiphase CECT (pre-contrast, arterial, venous, and delayed), Kim et al. [52] trained and tested a DL model using data from 1320 patients with either cirrhosis or chronic B virus hepatitis to detect HCC. The sensitivity varied according to size, with 33.3% for lesions <10 mm, 74.7% for those between 10–20 mm and 95.9% for lesions >20 mm, with an overall sensitivity of 84.8%. The most frequent cause of the error was an atypical enhancement pattern. Kim et al. [53] studied data from 549 patients with HCC who underwent MR imaging with gadoxetic acid (Gd-EOB-DTPA) to train and test a DL model for HCC detection. Using the hepatobiliary phase, the application had a sensitivity of 87%. Fabijańska et al. [54] obtained a sensitivity of 90.8% for HCC detection in cirrhotic patients. The DL model used integrated T1 dynamic acquisitions with extracellular contrast (non-contrast, arterial and late phase), but the dataset was small, with only nine patients. Integrating all three post-contrast acquisition phases proved superior to using either phase alone. An example of how a detection algorithm works is pictured in Figure 5, with bounding boxes (red) being applied to detected lesions.

**Table 2:** AI detection models.

Author	Year	Modality	AI-Method	Sensitivity
Tiyarattanachai et al. [50]	2022	US	DL (RetinaNet CNN)	89.8%
Lee et al. [51]	2019	CECT	DL (CNN)	93.8%
Kim et al. [52]	2021	CECT	DL (Mask R-CNN)	84.8%
Kim et al. [53]	2020	MRI	DL (CNN)	87%
Fabijańska et al. [54]	2018	MRI	DL (U-Net CNN)	90.8%

US (ultrasound), CECT (contrast-enhanced computed tomography), MRI (magnetic resonance imaging), DL (deep learning) CNN (convolutional neural network), R-CNN (region-based convolutional neural network).

### 4.2. Segmentation

Segmentation involves the labelling of pixels in an image to delineate with great precision a region of interest (e.g., lesions, viable tissue in tumour, organs, etc.). The gold standard is represented by manual segmentation done by radiologists. However, this is time-consuming and prone to inter-reader variability [55]. Therefore, the evaluation of segmentation performance is most often done using the Dice-Sørensen coefficient, with results varying between 0–1, with 1 meaning complete overlap.

Liver/liver lesion segmentation with CT represents the main interest regarding AI applications to hepatic imaging [21] as it shows great promise to optimize this process and provide fast, standardized segmentations. Some of the first grand challenges for liver segmentation were organised during the Medical Image Computing and Computer Assisted Intervention Conference (MICCAI) in 2007 [56] and 2008 [57], where only conventional ML methods were used. A shift was seen during the Liver Tumor Segmentation Challenge (LITS) in 2017 [58], where most applications were based on DL. The difficulty lies in the variable liver and liver lesion density and shape, similar densities with surrounding organs like the spleen, gastrointestinal tract, and heart, and the presence of artefacts. Furthermore, anatomical variants are common imaging findings, like accessory fissures or lobes, elongated left liver lobe and Riedel lobe [59]. With cirrhosis, the structure is even more heterogenous, and the contours are irregular, which makes segmentations even more difficult.

**Figure 6:**
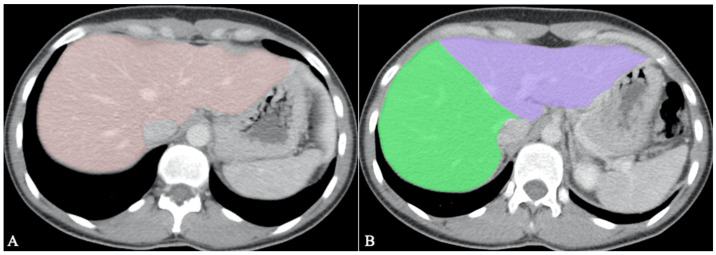
Volumetric measurements before LDLT; (**A**). Whole liver segmentation; (**B**). Right hemiliver/left hemiliver segmentation.

Hepatic segmentation is the preferred method for liver volumetry [55]. As living-donor liver transplantation (LDLT) becomes more widespread, it is mandatory to do volumetric evaluations before surgery, as inadequate graft volume is the main contraindication to LDLT [60]. An inaccurate transplanted liver size can cause a small-for-size syndrome with functional insufficiency, leading to death [55]. The minimum remnant liver volume for the adult population is 30%, provided there is no underlying liver dysfunction [61]. Therefore, the recipient’s ratio of graft size to standard liver volume according to body surface area should be over 40–50% [55,60]. An example of whole liver segmentation (A) and right-hemiliver/left-hemiliver segmentation (B) is provided in Figure 6.

The summary of the AI-segmentation models presented below is shown in Table 3.

Although there is great interest in developing DL models for hepatic segmentation, respecting the Couinaud [62] functional liver segmentation according to vascular supply is mandatory for clinical applications. Tian et al. [63] developed a DL method (GLC-UNet) to segment the liver according to Couinaud using 193 CT scans manually annotated by radiologists. The model obtained a DICE score of 92.46%. Wang et al. [64] used a cascaded neural network (ARH-CNet) to segment the liver according to Couinaud from 193 CT scans manually annotated by radiologists. The model obtained a DICE score of 84%. Using MR imaging, Han et al. [65] developed a 3D convolutional neural network on portal phase acquisitions from 744 scans. The average DICE score was 90.2%, and the dataset included cirrhotic patients. The authors also experimented with the localization of lesions according to segments with a 93.4% accuracy. 

**Figure 7:**
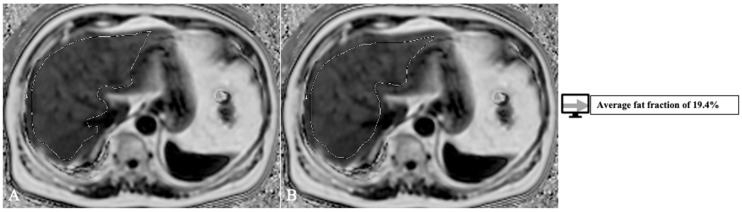
Exemplification of fat-fraction automatic quantification on MRI PDFF acquisitions with whole liver segmentation (1) at two different levels (**A**,**B**).

Another factor that influences transplant outcome is the presence of steatosis in the donor liver, which may lead to graft dysfunction and biliary and vascular complications [66]. The cut-off varies between 10 to 30% [60]. The gold standard for steatosis diagnosis is biopsy which only evaluates a tiny portion of parenchyma and is subject to inter-pathologist subjectivity [67]. MRI proton density fat fraction (PDFF) has been shown to have a very good diagnostic performance for liver fat assessment and grading [68], evaluating the whole liver structure. Thus, there is a need to develop AI models with more complex roles of both whole liver segmentation and fat quantification. Jimenez-Pastor et al. [69] developed a DL method on 183 MRI multi-echo chemical shift encoded (MECSE) liver studies with the ability to segment the liver and provide fat and iron quantifications. The DICE score for segmentation was 93%, and the model showed a high correlation and low relative error compared to manual fat and iron quantifications. An example of a model for PDFF segmentation and quantification is presented in Figure 7, with an analysis of the whole liver structure on multiple slices. 

**Figure 8:**
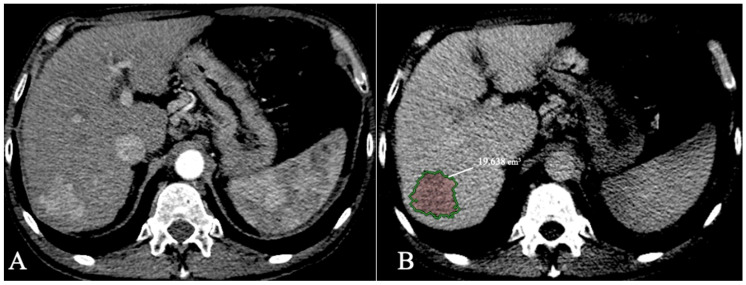
HCC with volumetric measurements; (**A**). Arterial phase; (**B**). Delayed phase segmentation.

HCC lesion segmentation with volumetric data extraction can also provide an aid to better select patients eligible for transplant with total tumour volume (TTV) as an inclusion criterion with a threshold of 115 cm^3^ [38]. Bousabarah et al. [70] analysed 174 patients with HCC scanned with MR imaging using a DL method (U-Net) to segment the liver and the lesions. The model used T1 postcontrast acquisitions (arterial, venous, and delayed) using extracellular agents and obtained a DICE score of 91% for liver segmentation and 68% for HCC segmentation. As volumetric assessment becomes automatic with an AI model, more precise and quantifiable inclusion criteria can be developed. For example, the LITS challenge [58] included a tumor burden metric as part of the AI algorithm segmentation accuracy evaluation (calculated as voxels of tumour/voxels of the liver). An example of HCC segmentation and volumetric measurements using CECT is provided in Figure 8. 

Automatic segmentation algorithms that help with transplant recipients can also be applied to assess sarcopenia, characterised by the loss of skeletal muscle mass and function. Sarcopenia impacts survival in the liver transplant setting as it is an independent predictor of orthotopic liver transplantation outcome [71], associated with higher mortality [72]. The quantitative assessment of body composition (defined as the percentage of muscle, fat, bone, and water) is usually done at the level of the third lumbar vertebrae [72] by segmentation of muscle, adipose tissue, and bone. AI can reduce segmentation times by providing automatic measurements and more standardised assessment techniques. Most of the research regarding sarcopenia evaluation using AI uses DL methods [73]. For example, blanc-Durand et al. [74] used a convolutional neural network and obtained a Dice score of 97% in a study done on 1025 CT scans.

**Table 3:** AI segmentation models.

Author	Year	Scope	Modality	AI-Method	DICE Score
Tian et al. [63]	2019	Couinaud segmentation	CECT	DL (GLC-UNet CNN)	92.46%
Wang et al. [64]	2022	Couinaud segmentation	CECT	DL (ARH-CNet CNN)	84%
Han et al. [65]	2022	Couinaud segmentation	MRI	DL (U-Net CNN)	90.2%
Jimenez-Pastor et al. [69]	2021	Liver segmentation, fat, and iron quantification	MRI	DL (CNN)	93%
Bousabarah et al. [70]	2021	Liver and HCC segmentation	MRI	DL (U-Net CNN)	91% for liver 68% for HCC
Durand et al. [74]	2020	Sarcopenia evaluation	CT	DL (U-Net CNN)	97%

CT (computed tomography), CECT (contrast-enhanced computed tomography), MRI (magnetic resonance imaging), HCC (hepatocarcinoma), DL (deep learning), CNN (convolutional neural network), GLC-UNet (global and local contexts UNet); ARH-CNet (attentive residual hourglass-based cascaded network).

### 4.3. Classification

#### 4.3.1. Microvascular Invasion

Microvascular invasion (MVI) is recognised as an essential factor for survival in patients with HCC after LT, and its presence doubles the risk of recurrence [33]. It is defined as tumour present in a vessel lined by endothelium seen by microscopy [75]. The ability to detect this feature before a transplant would allow for better transplant list stratification and risk assessment. The summary of the selected AI models to predict MVI is presented in Table 4.

Chen et al. [76] studied 415 patients with small HCC (<3 cm) from three independent institutions. They developed a radiomics signature from DCE MRI with an intracellular agent (Gd-EOB-DTPA) and DWI that predicted MVI with an AUC of 0.971. The HBP and DWI images were most relevant for MVI prediction. Jiang et al. [77] analysed 405 patients with HCC and triple-phase CT acquisitions (arterial, porto-venous, and delayed phase) using a 3D CNN with an AUC of 0.906. They compared these results with a radiomics model that included clinicopathological data, which obtained a lower AUC of 0.887. Sun et al. [78] used DL and AFP information to study 321 patients with HCC and DCE MR imaging with intracellular contrast (Gd-EOB-DTPA). They obtained an AUC of 0.824 in predicting MVI. An ablation study was done to demonstrate which is the most relevant acquisition for determining MVI. A combination of non-contrast T1, delayed, and porto-venous phases showed the best results, while DWI had less impact. Zhou et al. [79] analysed 114 patients undergoing MR imaging using extracellular contrast (Gd-DTPA) with T1, arterial and venous phases. The data from all three contrast acquisitions was processed using a 3D CNN that obtained an AUC for predicting an MVI of 0.926. They also tested the acquisition phases separately and observed that the arterial phase had the best performance (AUC of 0.855). 

#### 4.3.2. HCC Grading Prediction

Hepatocarcinoma grade is a biological marker for the aggressiveness of tumors, and, like MVI, it is an important prognostic indicator of recurrence for transplanted patients [80]. The most common classification is the Edmondson and Steiner (ES) according to the degree of differentiation (from well to undifferentiated) [81]. In transplant patients, a biopsy is indicated for excluding undifferentiated and poorly differentiated HCC in the Hangzhou criteria for lesions >8 cm, AFP < 400 ng/mL, and the Toronto Criteria for lesions beyond Milan. This marker can impact prognosis and allow for better patient stratification, as size, and histopathological differentiation are significant independent factors for survival [80]. The summary of selected AI models with HCC grading prediction functions is presented in Table 5.

Mao et al. [82] analysed 297 patients with HCC to develop a radiomics model that classifies lesions according to ES into low-grade or high-grade. CECT imaging data from dual-phase acquisitions (arterial and venous) and clinicopathological data were processed, and the application reached an AUC of 0.801. Even though arterial phase features showed more relevance for the prediction task, using both arterial and venous phases proved superior. Using non-contrast MR imaging and clinical data, Wu et al. [83] studied 170 patients with HCC to train a radiomics model that classifies lesions according to ES grade into low or high. The imaging protocol consisted of non-contrast T1 and T2 weighted images combined with clinical data and obtained an AUC of 0.8. Compared to a model that relied on imaging alone, the combined clinico-radiological model proved superior (0.742 versus 0.800). Han et al. [84] developed a combined clinical and imaging radiomics model to assess HCC grade using hepatospecific DCE MRI (Gd-EOB-DTPA) with T1-weighted, T2-weighted, hepatobilliary and portovenous imaging. The model analysed 137 patients with the hepatobiliary phase having the most significant impact on prediction, obtaining the highest AUC of 0.8. Zhou et al. [85] developed a model to predict ES grading on DWI MR images using a 3D CNN on a cohort of 98 patients obtaining an AUC of 0.83. MR acquisitions consisted of DWI using 0, 100 and 600 s/mm^2^ b-values and generated ADC maps. The highest b-values proved to be more valuable for classification. Zhou et al. [86] used a deep neural network (SE-DenseNet) to grade HCC lesions using the ES system from DCE MR images from a dataset of 75 patients. They used arterial, venous, and delayed phase images and obtained an AUC of 0.83. They focused more on comparing their model to other neural networks like DenseNet, ResNet and AlexNet, which performed worse.

#### 4.3.3. Molecular Evaluation

Several immunohistochemical markers can offer further information regarding prognosis or improve the positive diagnosis of HCC. Unfortunately, these can only be obtained from biopsy specimens or resected lesions. The summary of selected AI models that could be used for molecular evaluation is presented in Table 6.

Glypican 3 (GPC3) is present on the cell surface and has been included in the panel of markers for HCC diagnosis in highly differentiated small lesions [26]. It can also act as a marker of poor prognosis [87]. The presence of GPC3 in HCC lesions impacts survival as it has been associated with a higher incidence of MVI [88], a reduced 5-year survival rate and disease-free survival in patients with LT [88,89]. Gu et al. [90] analysed a cohort of 293 patients with HCC that underwent MR imaging and developed a radiomics model based on clinical and imaging data to predict the presence of GPC3. MR protocol consisted of T1-weighted postcontrast acquisitions using extracellular contrast (Gd-DTPA) with arterial, venous, and delayed phases. The model that used only imaging data obtained an AUC of 0.871, and when combined with AFP, the AUC increased to 0.914. In a recent study, Chong et al. [91] studied 259 patients with HCC that underwent MR imaging with intracellular contrast (Gd-EOB-DTPA) to develop a radiomics model that predicts the presence of GPC3. The study showed that the most relevant imaging features were from T2 weighted images and T1 hepatobiliary phase, and together with clinical data, a nomogram was created that obtained an AUC of 0.943.

Cytokeratin 19 (CK19) is normally expressed in hepatic progenitor cells but not in healthy hepatocytes, and its presence in HCC lesions is a marker of aggressiveness and poor prognosis [26,27]. In patients with HCC that have undergone transplants beyond Milan criteria, CK19 has been associated with recurrence. In contrast, patients without expression of CK19 showed similar survival rates as those within the Milan criteria [92]. Zhang et al. [93] studied 214 patients and developed a radiomics model using ultrasound imaging and clinical data to predict the presence of CK19. The combined model obtained an AUC of 0.867, while the one that used only imaging data had a lower AUC of 0.789, showing the relevance of combining multiple types of input data. Yang et al. [94] analysed 257 patients with HCC from multiple centres that underwent MR imaging and developed a radiomics model to determine the presence of CK19+ lesions. The model with the best predictive performance used features from T2 and DWI with an AUC of 0.790. Chen et al. [95] developed a radiomics model using data from 80 patients with HCC to determine the presence of CK19. The imaging protocol consisted of MR imaging with intracellular contrast (Gd-EOB-DTPA), and the data was obtained from two institutions. They obtained an AUC of 0.833 by adding clinical data like AFP to improve performance while relying on imaging alone resulted in an AUC of 0.82. Analysing the data, they found that targetoid features on imaging were correlated with the presence of CK19.

**Table 6:** AI models for molecular evaluation.

Author	Year	Scope	Data	AI-Method	AUC
Gu et al. [90]	2020	GPC3 prediction	DCE-MRI (Gd- DTPA) + Clinical	Radiomics	0.914
Chong et al. [91]	2023	GPC3 prediction	DCE-MRI (Gd-EOB-DTPA) + Clinical	Radiomics	0.943
Zhang et al. [93]	2022	*CK19* prediction	US + Clinical	Radiomics	0.867
Yang et al. [94]	2021	*CK19* prediction	DCE-MRI (Gd-EOB-DTPA)	Radiomics	0.79
Chen et al. [95]	2021	*CK19* prediction	DCE-MRI (Gd-EOB-DTPA) + Clinical	Radiomics	0.833

MRI (magnetic resonance imaging), DCE-MRI (dynamic contrast-enhanced magnetic resonance imaging), Gd-DTPA (gadolinium diethylenetriamine penta-acetic acid), Gd-EOB-DTPA (gadolinium ethoxybenzyl-diethylenetriamine penta-acetic acid), US (ultrasound).

## 5. Discussion and Limitations

Multiple applications of AI can be used in the setting of liver transplants. These range from the initial detection, which might enhance the ability to identify smaller lesions, to automatically segmenting liver and liver lesion volumes. Other non-liver imaging markers that influence liver transplant outcomes, such as body composition volumes, can also be automatically evaluated from the same study. Furthermore, the in-depth pixel analysis of lesions could provide imaging markers that are impossible to assess with the human eye. All these AI tasks (detection, segmentation, classification) would work as automatic processing steps that could impact survival, such as early detection, sarcopenia, and the presence of imaging markers (MVI, grading, GPC3, CK19) are essential for prediction outcomes. Some of these markers can only be obtained by biopsy pretransplant, which is prone to sampling errors and has a low sensitivity and positive predictive value in the accurate classification of HCC grade and low concordance with explant pathology [96]. In this context, tools that can analyse the entire tumour structure using multimodality imaging might provide more reliable information before transplant. The individual assessment of characteristics like grading, MVI or the presence of CK19 and GPC3 could be combined in a complex model for risk assessment since some of these markers have common imaging features. For example, qualitative evaluation of rim arterial enhancement and irregular margins can be associated with both MVI [97] and the presence of CK19 [98], while T1 hypointensity is associated with both low-grade lesions [99] and MVI [97]. Similarly, reduced hepatobiliary phase intensity can be found in low-grade lesions [99] and CK19+ [98]. 

Multiple types of imaging data were used to develop these models. Since the US is the most widely used technique for screening, it should be a priority for AI detection algorithms, especially in the setting of chronic liver disease. For segmentation, CT is more often used for model development, which is expected since it is more readily available and cheaper than MRI in clinical practice, although radiation exposure must be considered. The in-depth analysis on a pixel level using DL and radiomics was most often applied to MRI data, using non-contrast sequences [83,85], extracellular [79,86,90] and intracellular contrast [76,78,84,91,94,95]. This can be explained by using multiple types of sequences, and contrast agents in MRI imaging can provide more information than CT or US.

The ideal scenario for developing AI applications is to integrate multiple functions in one model that could help transplant patients. The robustness of AI models means that multiple data types can be integrated, including patient demographics, clinical and genetic data, lab values and imaging markers. For example, some radiomics models analyze clinical and imaging data for output generation [76,78,82,83,90,91,93,95], and ablation studies comparing the results show better accuracy when using multiple types of data [83,90,93,95]. In addition, other more serologic biomarkers are evaluated in the setting of HCC, like the type of circulating nucleic acid, which could help in early diagnosis and prediction of response to treatment [100].

One barrier to developing these models is the lack of public datasets. For segmentation, there are some public datasets available for CT, like the LITS [56] and 3DIRCAD [101] but only CHAOS [102] for MRI. Increasing the amount of training data while providing multi-centre acquisitions can boost the performance of AI models [103]. These multi-centre datasets must be heterogenous enough to eliminate biases relating to race, gender, ethnicity, and age. Regarding the development of biological markers like MVI, GLY3, CK19 or recurrence prediction, all studies were done retrospectively, and no public databases exist. Open competitions on common datasets would allow better comparison between models so that the performance can be evaluated consistently. This would also permit the evaluation of models and different techniques like deep learning versus radiomics. While DL allows for a more end-to-end evaluation, it does not give sufficient data on the selected features. On the other hand, radiomics needs more human input but enables the user to see what feature is most relevant. A meta-analysis of 16 studies for predicting MVI preoperatively showed high diagnostic accuracy for AI applications and better performance for DL versus non-DL models [104]

AI models are frequently reported as “black boxes” because even though they provide predictive answers, at least for DL, no explanation for these outputs exists. Therefore, the question of why some predictions is made remains unanswered. Some authors that used multiple types of imaging data for model development have assessed the impact each sequence or contrast phase has on the final result [76,78,79,82,84,85,91,94,95] or the relevance of combining features from multiple acquisitions [82]. Another way which might provide more information for the radiologist is the development of feature maps highlighting the region of interest that impacted the result most, like the impact of targetoid enhancement or diffusion restriction on the prognosis of CK19 presence [95]. Thus, having a transparent feature selection process that radiologists can evaluate might help bridge the gap between clinicians and AI models. In response, the concept of Explainable Artificial Intelligence (XAI) [105] has emerged to ensure models are performant and that the imaging features that have the most impact on the decision can be highlighted for further study.

For the development of reproducible and transparent models, authors have to provide enough details to allow for full comprehension of the scope and methods used. Therefore, some AI-adapted publishing guidelines, like CLAIM (Checklist for Artificial Intelligence in Medical Imaging) [106], have been made available for authors and reviewers to ensure quality standards are met.

## 6. Conclusions

A liver transplant is an ideal treatment for patients with hepatocellular carcinoma and advanced chronic liver disease. Still, the lack of organ availability favours the adoption of rigorous selection criteria for candidates. In this setting, artificial intelligence can provide an aid to extract more quantitative data from imaging and integrate these features with clinical data to develop complex models that process large amounts of information. Thus, they can offer better assessments of liver transplant candidates to ensure the best survival rates and reduce the recurrence of HCC. However, even with promising results, these models must be validated in a prospective clinical setting, and the issue of limited public datasets should be addressed. 

## Figures and Tables

**Table 1:** LT criteria.

CRITERIA	REPORT
MILAN [25]	One lesion ≤5 cm or a maximum of 3 lesions each ≤3 cm
University of California, San Francisco (UCSF) [32]	One lesion ≤6.5 cm or a maximum of 3 lesions with the largest tumor diameter ≤4.5 cm and a total tumor diameter ≤8
Up-to-7 [33]	The sum of the number of lesions and the diameter of the largest lesion ≤7
Updated Up-to-7/Metroticket V2.0 [34]	A combination of the sum of the number of lesions, the largest lesion diameter and AFP
AFP model [35]	A score based on the largest tumour diameter, number of nodules and AFP; A result of ≤2 is an indication of a transplant
UNOS criteria [36]	One lesion ≥2 cm and ≤5 cm or maximum 3 lesions each ≥1 cm and ≤3; AFP ≤1000 ng/dl
Extended Toronto [37]	No tumour size and number limit; Biopsy needed beyond Milan to exclude poorly differentiated
Total tumor volume (TTV) [38]	TTV of less than 115 cm^3^
Hangzhou criteria [39]	Total tumor diameter ≤8 cm or >8 cm with histopathologic grade 1 or 2 and a preoperative AFP value of ≤400
TRAIN score [40]	mRECIST response; AFP slope; Neutrophil-to-lymphocyte ratio (NLR) and platelet-to-lymphocyte ratio (PLR); Waitlist time

**Table 4:** AI models to predict MVI.

Author	Year	Scope	Data	AI-Method	AUC
Chen et al. [76]	2022	MVI prediction	DCE-MRI (Gd-EOB-DTPA) + Clinical	Radiomics	0.971
Jiang et al. [77]	2021	MVI prediction	CECT	DL (CNN)	0.906
Sun et al. [78]	2022	MVI prediction	DCE-MRI (Gd-EOB-DTPA) + Clinical	DL (ResNet CNN)	0.824
Zhou et al. [79]	2021	MVI prediction	DCE-MRI (Gd- DTPA)	DL (CNN)	0.926

MRI (magnetic resonance imaging), CECT (contrast-enhanced computed tomography), Gd-EOB-DTPA (gadolinium ethoxy benzyl-diethylenetriamine penta-acetic acid), Gd-DTPA (gadolinium diethylenetriamine penta-acetic acid), DL (deep learning), CNN (convolutional neural network).

**Table 5:** AI models with HCC grading functions.

Author	Year	Scope	Data	AI-Method	AUC
Mao et al. [82]	2020	Grading prediction	CECT + Clinical	Radiomics	0.801
Wu et al. [83]	2019	Grading prediction	MRI + Clinical	Radiomics	0.8
Han et al. [84]	2023	Grading prediction	DCE MRI (Gd-EOB-DTPA)	Radiomics	0.8
Zhou et al. [85]	2019	Grading prediction	MRI	DL (CNN)	0.83
Zhou et al. [86]	2019	Grading prediction	DCE MRI (Gd- DTPA)	DL (DenseNet CNN)	0.83

CECT (contrast-enhanced computed tomography), MRI (magnetic resonance imaging), DWI (diffusion-weighted imaging), Gd-DTPA (gadolinium diethylenetriamine penta-acetic acid), Gd-EOB-DTPA (gadolinium ethoxybenzyl-diethylenetriamine penta-acetic acid), DL (deep learning), CNN (convolutional neural network).

## Data Availability

Images included in the present article were obtained retrospectively from Fundeni Clinical Institute.
